# Supplementary material for: Effect of Drought and Heavy Metal Contamination on Growth and Photosynthesis of Silver Birch Trees Growing on Post-Industrial Heaps
Source: Cells. 2021 Dec 24;11(1):53. doi: 10.3390/cells11010053 (PMC8750922; doi:10.3390/cells11010053)
Supplement: Supplementary file 1 [file cells-11-00053-s001.zip › cells-1512371-supplementary.pdf]

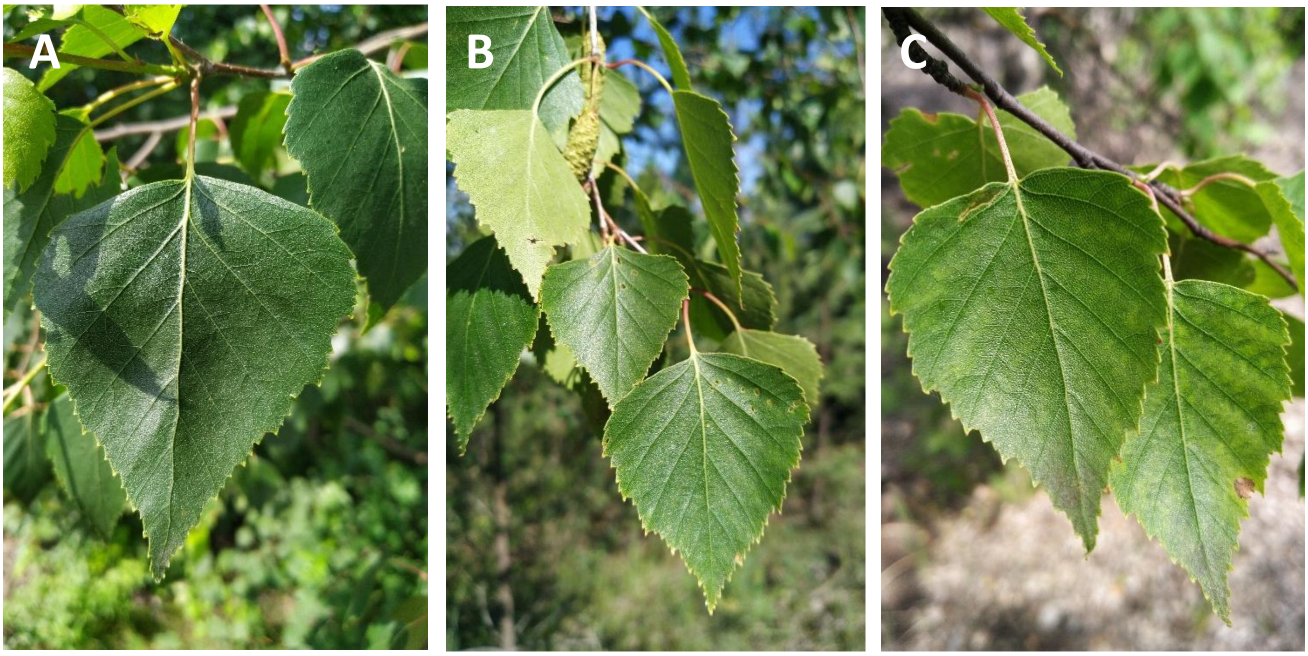

Figure S1. Photographs of typical birch leaves from reference site in Tychy (A), post-coal mine heap in Katowice (B) and post-smelter heap in Piekary Śląskie (C).

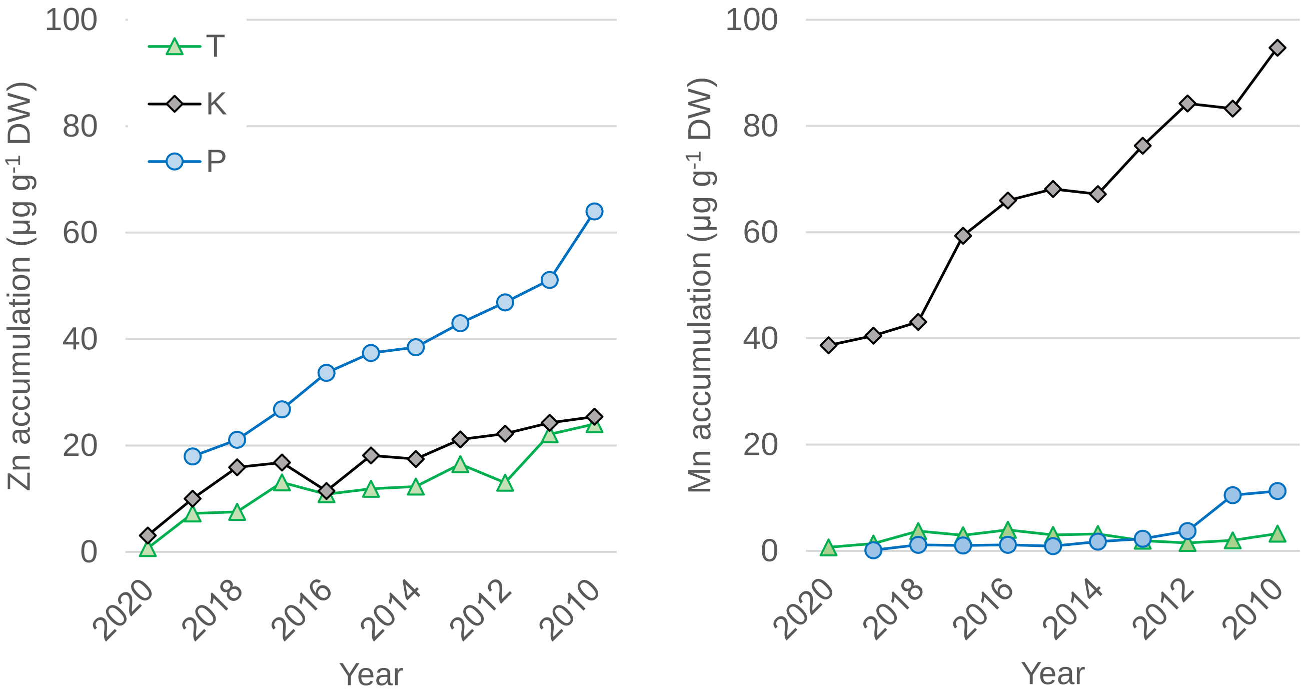

Figure S2. The accumulation of zinc or manganese in birch wood from a particular year of growth. Data are means (n = 10). Abbreviations: T – reference site in Tychy; K – post-coal mine heap in Katowice; P – post-smelter heap in Piekary Śląskie.

Table S1. Site and soil characteristics.

Site characteristics			
	T	K	P
Location	Tychy	Katowice-Murcki	Piekary Śląskie
Altitude (m AMSL)	259	321	287
Temperature (°C)[a]	9.0	9.1	9.1
Precipitation (mm)[a]	817	807	910
Latitude	50.1043268	50.1895495	50.3681323
Longitude	18.9644241	19.0322035	18.9689818
Habitat	suburban area	post-coal mine spoil heap	post-smelter spoil heap
Soil characteristics			
pH (H_2O)	6.09 ± 0.15 b	3.68 ± 0.06 c	7.33 ± 0.08 a
EC (µS cm^{-1})	160 ± 28 b	113 ± 10 b	800 ± 91 a
Total element content in the soil (µg g^{-1} DW)			
Ca	2140 ± 440 b	160 ± 40 c	81070 ± 3420 a
Cd	0.52 ± 0.07 b	BDL	212.51 ± 34.90 a
Cu	7.2 ± 2.0 c	28.3 ± 0.9 b	961.5 ± 131.8 a
Fe	5160 ± 300 b	6160 ± 150 a	6890 ± 410 a
K	2340 ± 570 b	9560 ± 370 a	670 ± 110 c
Mg	1070 ± 210 b	910 ± 50 b	16920 ± 1920 a
Mn	350 ± 40 b	160 ± 10 c	3900 ± 190 a
Zn	25.0 ± 3.0 c	138.7 ± 26.8 b	25545.1 ± 4110.0 a

[a] – data from climate-data.org

Abbreviations: T – reference site in Tychy; K – post-coal mine heap in Katowice; P – post-smelter heap in Piekary Śląskie.

Table S2. Element accumulation in bark and wood of studied birch populations.

	T	K	P
Element accumulation in bark (µg g^{-1} DW)			
Ca	4915 ± 52 b	563 ± 63 c	7542 ± 420 a
Cd	BDL	BDL	3.02 ± 0.36 a
Cu	BDL	BDL	BDL
Fe	74.29 ± 0.91 a	24.28 ± 1.12 b	26.92 ± 2.48 b
K	462 ± 18 a	46 ± 4 c	265 ±12 b
Mg	166 ± 13 b	148 ± 15 b	696 ± 27 a
Mn	38.8 ± 2.4 b	257.5 ± 14.4 a	15.9 ± 1.5 c
Zn	113.5 ± 5.5 b	92.3 ± 6.2 b	230.5 ± 12.4 a
Element accumulation in wood (µg g^{-1} DW)			
Ca	382 ± 40 b	188 ± 35 c	923 ± 91 a
Cd	BDL	BDL	4.44 ± 0.57 a
Cu	BDL	BDL	BDL
Fe	50.28 ± 4.98 a	25.26 ± 3.39 b	14.62 ± 5.52 b
K	BDL	BDL	BDL
Mg	100 ± 18 b	129 ± 9 b	335 ± 35 a
Mn	3.7 ± 0.5 b	96.0 ± 2.8 a	1.0 ± 0.2 b
Zn	12.0 ± 0.8 c	25.5 ± 1.9 b	45.6 ± 3.1 a

Abbreviations: T – reference site in Tychy; K – post-coal mine heap in Katowice; P – post-smelter heap in Piekary Śląskie. BDL – below detection limit. Data are means ± SE (n = 10).

Table S3. The correlation matrix of variables used in the PCA.

	LEF	NPQ_T	Φ_{II}	Φ_{NO}	Φ_{NPQ}	PS1AC	Thick	E	A	ci	Chl	Flav	Anth	Ca	Mg	Mn	Zn	TRW
LEF	**1.00**	-0.20	0.11	0.18	-0.28	0.17	-0.58	0.61	0.32	0.04	0.31	-0.59	-0.46	0.15	-0.42	-0.31	-0.07	-0.12
NPQ_T	**-0.20**	**1.00**	-0.68	-0.61	0.94	-0.14	0.45	-0.06	-0.11	0.07	-0.27	0.44	0.31	-0.31	-0.05	0.59	-0.25	0.52
Φ_{II}	**0.11**	**-0.68**	**1.00**	-0.08	-0.79	0.37	-0.56	0.19	0.36	-0.24	0.31	-0.30	-0.39	0.28	-0.10	-0.44	0.09	-0.28
Φ_{NO}	**0.18**	**-0.61**	**-0.08**	**1.00**	-0.47	-0.22	-0.05	-0.11	-0.19	0.10	0.01	-0.21	0.01	0.17	0.16	-0.26	0.22	-0.30
Φ_{NPQ}	**-0.28**	**0.94**	**-0.79**	**-0.47**	**1.00**	-0.20	0.60	-0.19	-0.27	0.19	-0.36	0.53	0.41	-0.37	0.07	0.62	-0.20	0.48
PS1 AC	**0.17**	**-0.14**	**0.37**	**-0.22**	**-0.20**	**1.00**	-0.31	0.35	0.44	-0.23	0.42	-0.36	-0.51	-0.13	-0.25	-0.14	-0.14	0.03
Thick	**-0.58**	**0.45**	**-0.56**	**-0.05**	**0.60**	**-0.31**	**1.00**	-0.52	-0.48	0.21	-0.40	0.71	0.57	-0.28	0.27	0.68	-0.06	0.43
E	**0.61**	**-0.06**	**0.19**	**-0.11**	**-0.19**	**0.35**	**-0.52**	**1.00**	0.85	-0.42	0.49	-0.66	-0.61	-0.06	-0.64	-0.07	-0.40	0.24
A	**0.32**	**-0.11**	**0.36**	**-0.19**	**-0.27**	**0.44**	**-0.48**	**0.85**	**1.00**	-0.66	0.58	-0.55	-0.63	-0.02	-0.59	-0.07	-0.43	0.25
ci	**0.04**	**0.07**	**-0.24**	**0.10**	**0.19**	**-0.23**	**0.21**	**-0.42**	**-0.66**	**1.00**	-0.58	0.42	0.60	0.08	0.69	-0.12	0.44	-0.43
Chl	**0.31**	**-0.27**	**0.31**	**0.01**	**-0.36**	**0.42**	**-0.40**	**0.49**	**0.58**	**-0.58**	**1.00**	-0.50	-0.79	-0.11	-0.58	-0.07	-0.44	0.24
Flav	**-0.59**	**0.44**	**-0.30**	**-0.21**	**0.53**	**-0.36**	**0.71**	**-0.66**	**-0.55**	**0.42**	**-0.50**	**1.00**	0.74	-0.18	0.49	0.52	0.12	0.22
Anth	**-0.46**	**0.31**	**-0.39**	**0.01**	**0.41**	**-0.51**	**0.57**	**-0.61**	**-0.63**	**0.60**	**-0.79**	**0.74**	**1.00**	0.07	0.67	0.27	0.37	-0.07
Ca	**0.15**	**-0.31**	**0.28**	**0.17**	**-0.37**	**-0.13**	**-0.28**	**-0.06**	**-0.02**	**0.08**	**-0.11**	**-0.18**	**0.07**	**1.00**	0.32	-0.51	0.77	-0.58
Mg	**-0.42**	**-0.05**	**-0.10**	**0.16**	**0.07**	**-0.25**	**0.27**	**-0.64**	**-0.59**	**0.69**	**-0.58**	**0.49**	**0.67**	**0.32**	**1.00**	-0.12	0.58	-0.47
Mn	**-0.31**	**0.59**	**-0.44**	**-0.26**	**0.62**	**-0.14**	**0.68**	**-0.07**	**-0.07**	**-0.12**	**-0.07**	**0.52**	**0.27**	**-0.51**	**-0.12**	**1.00**	-0.50	0.89
Zn	**-0.07**	**-0.25**	**0.09**	**0.22**	**-0.20**	**-0.14**	**-0.06**	**-0.40**	**-0.43**	**0.44**	**-0.44**	**0.12**	**0.37**	**0.77**	**0.58**	**-0.50**	**1.00**	-0.75
TRW	**-0.12**	**0.52**	**-0.28**	**-0.30**	**0.48**	**0.03**	**0.43**	**0.24**	**0.25**	**-0.43**	**0.24**	**0.22**	**-0.07**	**-0.58**	**-0.47**	**0.89**	**-0.75**	**1.00**

Gradient:
> 0.75
0.75 > 0.5
0.5 > 0.25
0.25 > -0.25
-0.25 > -0.5
-0.5 > -0.75
-0.75 >

Abbreviations: LEF – linear electron flow; NPQ_T – non-photochemical quenching; Φ_{II} – realized steady-state efficiency of PSII; Φ_{NO} – non-regulatory energy dissipation; Φ_{NPQ} – non-photochemical quenching; PS1 AC – PSI active centres; Thick – leaf thickness; E – transpiration rate; A – photosynthetic rate; ci – intracellular CO_2 content; Chl – chlorophyll content; Flav – flavonol content; Anth – anthocyanin content; Ca – calcium content in leaves; Mg – magnesium content in leaves; Mn – manganese content in leaves; Zn – zinc content in leaves; TRW – tree ring width.
